# Morphological and biomechanical disparity of crocodile-line archosaurs following the end-Triassic extinction

**DOI:** 10.1098/rspb.2013.1940

**Published:** 2013-11-07

**Authors:** Thomas L. Stubbs, Stephanie E. Pierce, Emily J. Rayfield, Philip S. L. Anderson

**Affiliations:** 1 School of Earth Sciences, University of Bristol, Wills Memorial Building, Queen's Road, Bristol BS8 1RJ, UK; 2 Structure and Motion Laboratory, Department of Comparative Biomedical Sciences, The Royal Veterinary College, Hawkshead Lane, Hatfield AL9 7TA, UK; 3 Department of Biology, Duke University, Box 90338, Durham, NC 27708, USA

**Keywords:** crurotarsan, disparity, evolutionary radiation, ecological diversity, form and function, mandible

## Abstract

Mesozoic crurotarsans exhibited diverse morphologies and feeding modes, representing considerable ecological diversity, yet macroevolutionary patterns remain unexplored. Here, we use a unique combination of morphological and biomechanical disparity metrics to quantify the ecological diversity and trophic radiations of Mesozoic crurotarsans, using the mandible as a morpho-functional proxy. We recover three major trends. First, the diverse assemblage of Late Triassic crurotarsans was morphologically and biomechanically disparate, implying high levels of ecological variation; but, following the end-Triassic extinction, disparity declined. Second, the Jurassic radiation of marine thalattosuchians resulted in very low morphological disparity but moderate variation in jaw biomechanics, highlighting a hydrodynamic constraint on mandibular form. Third, during the Cretaceous terrestrial radiations of neosuchians and notosuchians, mandibular morphological variation increased considerably. By the Late Cretaceous, crocodylomorphs evolved a range of morphologies equalling Late Triassic crurotarsans. By contrast, biomechanical disparity in the Cretaceous did not increase, essentially decoupling from morphology. This enigmatic result could be attributed to biomechanical evolution in other anatomical regions (e.g. cranium, dentition or postcranium), possibly releasing the mandible from selective pressures. Overall, our analyses reveal a complex relationship between morphological and biomechanical disparity in Mesozoic crurotarsans that culminated in specialized feeding ecologies and associated lifestyles.

## 1. Introduction

Crocodylomorphs are the only crurotarsan archosaurs (pseudosuchians) that survived the end-Triassic extinction (ETE) approximately 201 Ma. Their ecologically diverse relatives, the phytosaurs, ‘rauisuchians’, ornithosuchids and aetosaurs, all became extinct during this global event [1,2]. Continuing fossil discoveries reveal that Mesozoic crocodylomorphs had much greater morphological disparity (diversity of forms) when compared with modern crocodilians [3,4]. This disparity resulted from major adaptive diversifications, during which Mesozoic crocodylomorphs evolved lifestyles and feeding ecologies unlike anything seen today, including small cursorial insectivores, terrestrial and marine hypercarnivores, and highly specialized herbivores and marine piscivores [3–7]. The patterns and processes associated with the evolution of these divergent feeding modes, and the related structural and functional innovations, remain relatively unexplored. In particular, very few studies have used integrative, comparative and quantitative analyses to assess this observed ecological diversity [6,8].

Previous research into the diversification of Mesozoic crocodylomorphs has focused on crurotarsan morphological disparity across the Triassic–Jurassic boundary (TJB) based on variation in cladistic (phylogenetic) characters [9,10]. Results from cranial characters suggest disparity did not change significantly across the TJB, despite the extinction of multiple ecologically diverse groups, and that morphological variation of Early Jurassic crocodylomorphs accumulated rapidly [10]. In addition to cladistic characters, morphological disparity can also be quantified using geometric morphometric landmarks, allowing structural changes in forms to be observed [11,12]. When interpreting ecological diversifications based on both cladistic characters and geometric morphometrics, it is assumed that variation in morphology directly reflects variation in ecology [13,14]. However, morphological disparity can be disassociated from ecologically relevant biomechanical variables [13–18]. Biomechanical disparity is an additional complementary metric that quantifies variation based on characters within the musculoskeletal system that have known biomechanical significance [19,20].

As a homologous unit, the mandible is particularly well suited to geometric morphometric and biomechanical character analyses. Mandibular elements also have fundamental adaptive significance because their primary role is to capture, manipulate and process materials during feeding [14,20,21]. Although the upper jaws and crania also contribute to feeding innovations, and an organism's ecology is linked to its whole morphology, these structures are replete with trade-offs (e.g. sensory organs and nervous system). Additionally, complete mandibles are composed of fewer elements than complete skulls and are more likely to be preserved in their entirety, providing a significantly greater sample size.

Here, we examine the diversification of Mesozoic crocodylomorph feeding ecologies by quantifying morphological and biomechanical disparity in the mandible. We aim to identify periods of significant morphological and biomechanical evolution and track the ecological divergence of major taxonomic assemblages, using the mandible as a proxy. These evolutionary radiations are placed in the context of ecological parameters, such as diet and habitat, to determine whether they acted as constraints or stimuli for major innovations and diversifications. The degree to which our two metrics of disparity covary is assessed. We also aim to independently examine changes to crurotarsan disparity across the TJB, by conducting analyses of both morphological and biomechanical variation in the mandible incorporating Late Triassic non-crocodylomorph crurotarsans.

## 2. Material and methods

### (a) Taxon sampling

The morphological and biomechanical database assembled for this study includes 107 mandibular specimens representing 102 species (23 non-crocodylomorph crurotarsans and 79 crocodylomorphs; see the electronic supplementary material, table S1). Sampling was taken at species level to increase the sample size and accommodate intrageneric variation. For instances where specimens of the same species displayed significant intraspecific variation, specimens were treated as separate samples of the same species and both included.

### (b) Groupings

Monophyletic groups, evolutionary grades and non-monophyletic assemblages were used to generate comparative taxonomic groupings, reflecting uncertainties in crurotarsan interrelationships. The non-crocodylomorph crurotarsans were grouped as Phytosauria, Aetosauria and ‘other’ non-crocodylomorph crurotarsans (including ‘rauisuchians’, ornithosuchids and *Erpetosuchus*). The crocodylomorphs were grouped as ‘Sphenosuchia’, ‘protosuchians’ (including *Hsisosuchus*), Teleosauridae (Thalattosuchia), Metriorhynchidae (Thalattosuchia), Notosuchia, peirosaurids and mahajangasuchids, pholidosaurids and stomatosuchids, ‘other’ neosuchians and Eusuchia (see extended discussion in the electronic supplementary material, table S2).

Specimens were also partitioned according to interpreted diet and mode of life/habitat. The dietary groups are: small carnivores/insectivores (less than 10 cm mandible length), medium-sized carnivores/generalists (10–30 cm mandible length), large carnivores (more than 30 cm mandible length), piscivores and facultative herbivores. The modes of life/habitats are: marine, semiaquatic, terrestrial and putatively fossorial. Classifications are based on reports and discussions from the literature (see extended discussion in the electronic supplementary material, tables S3 and S4).

### (c) Stratigraphic binning

The taxa included in this study range temporally from the Carnian to the Maastrichtian. This timespan was divided into six epoch-level time bins: Late Triassic, Early Jurassic, Middle Jurassic, Late Jurassic, Early Cretaceous and Late Cretaceous [22]. Epoch-level time bins are selected over narrower stage-level time intervals to avoid under-population. For a higher resolution analysis of the TJB, the Late Triassic was separated into the Carnian and Norian–Rhaetian, and the Early Jurassic was separated into the Hettangian–Sinemurian and Pliensbachian–Toarcian (see the electronic supplementary material, table S5). Species-level stratigraphic ranges were used to assign specimens to time bins, and all assignments are derived from the literature.

### (d) Landmarks and morphometrics

Geometric morphometrics was implemented to calculate and visualize mandibular morphological variation. Shape variation in lateral profile was quantified using two-dimensional ‘type 2’ landmarks [23]. Six fixed landmarks were developed and positioned on discrete morphological features. To incorporate variation arising from curvature and to capture the overall shape of the mandible, 68 semi-landmarks were added along four curves positioned on the lateral outline of the mandible [23] (see the electronic supplementary material, table S6 and figure S1). This brought the total number of landmarks to 74. Landmark coordinates were superimposed using generalized least-squares Procrustes methods, removing the effects of orientation, positioning and scale. The corrected Procrustes coordinates were subjected to principal components (PC) analysis. The first two axes, representing the majority of morphological variation (PC1–46.3% and PC2–14.5%), were plotted to assess shape variation and produce a morphospace (see the electronic supplementary material, table S9 and figure S13). Variation in morphospace occupancy between stratigraphic intervals was assessed using a series of non-parametric multivariate analysis of variance (NPMANOVA). In all statistical analyses in this study, significance values are corrected for multiple comparisons using the false discovery rate procedure [24]. For a list of software used, see the electronic supplementary material.

### (e) Biomechanical analysis

To calculate biomechanical disparity, and produce a biomechanical morphospace, 14 relevant characters were measured from photographs and figures of mandibular specimens. The characters are based on simple lever mechanics, ratios and linear measurements, such as mechanical advantage, second moment of area and the quadrate–articular offset. Each character has known biomechanical consequences and together they characterize the emergent functional properties of the mandible [13,14,20] (see the electronic supplementary material, section 7). All measurements were normalized using the *z* transformation, so each character had an average value of zero. The normalized biomechanical character matrix was subjected to principal coordinates analysis (PCO) to ordinate taxa and produce a biomechanical morphospace, based on the first two axes representing the highest proportion of variation (PC1—18.0% and PC2—11.5%) (see the electronic supplementary material, table S10 and figure S14). The strength of association between each biomechanical character and the coordinate axes was tested using the Pearson correlation coefficient (see the electronic supplementary material, table S11). The dataset included both normally and non-normally distributed data, so some values represent approximations. PCO was selected as the appropriate analytical technique for the biomechanical dataset as it can be computed with missing data (the biomechanical dataset is 82% complete). Differences between centroid positions for each time period were assessed using a series of NPMANOVAs. For a list of software used, see the electronic supplementary material.

### (f) Disparity

Morphological and biomechanical disparity in each time bin was calculated based on the first 10 coordinate axes expressing the highest proportions of variance (see the electronic supplementary material, tables S9 and S10). The sum of variances metric is plotted and selected for discussion as it is robust to uneven sampling and outliers [25], but other disparity metrics return the same trends (see the electronic supplementary material, figures S15–S18). Bootstrapping was implemented to produce 95% CIs by resampling the 10 coordinate axes and calculating disparity with 1000 repetitions. The significance of changes in disparity through time was assessed using a series of pairwise *t*-tests. Marginal likelihoods for variance between time bins were also computed as alternative tests for changes in disparity, following the procedure of Finarelli & Flynn [26]. Likelihood ratios (LRs) were examined to determine whether changes in disparity between successive intervals were significant [20,26]. Partial disparity was calculated to examine the relative contribution of major taxonomic, dietary and habitat groups to overall morphological and biomechanical disparity in each stratigraphic interval [27]. For a list of software used, see the electronic supplementary material.

## 3. Results

### (a) Trends of disparity

**Figure 1.**
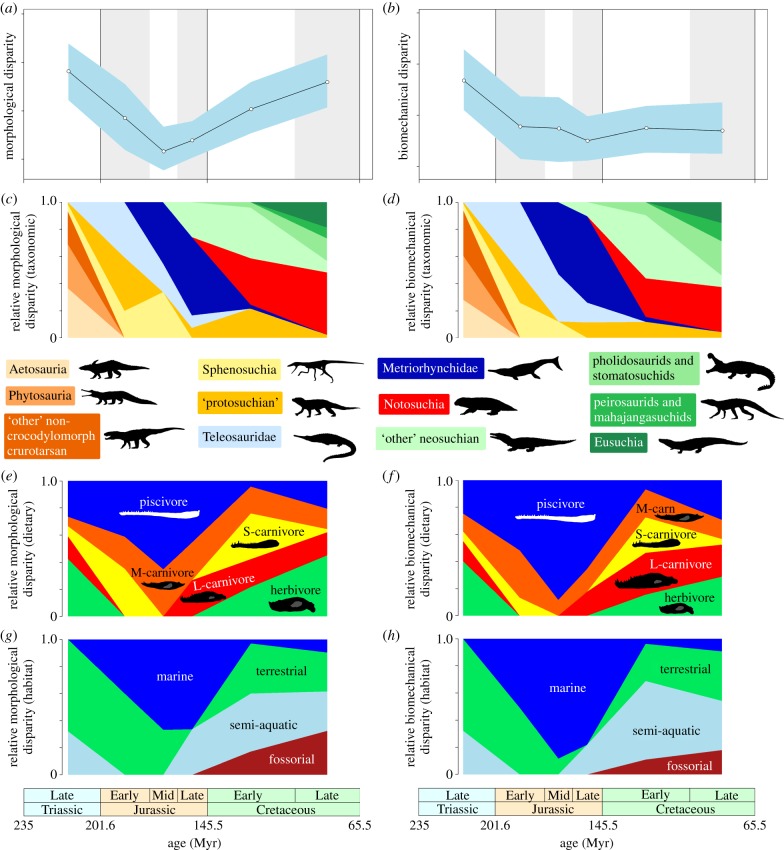
Morphological and biomechanical disparity for Mesozoic crurotarsan mandibles. Morphological (*a*) and biomechanical (*b*) disparity (sum of variances) are plotted in six time bins: Late Triassic, Early Jurassic, Middle Jurassic, Late Jurassic, Early Cretaceous and Late Cretaceous. The shaded components represents 95% CIs based on 1000 bootstrap pseudoreplicates. Partial disparity metrics (*c*–*h*) were also calculated to assess the relative contributions of various *ad hoc* subgroups, including taxonomic (*c*,*d*), dietary (*e*,*f*) and habitat (*g*,*h*) groups, where S, small; M, medium and L, large carnivore. All horizontal axes represent time, based on the scales provided. For non-rarefied results and alternative disparity metrics, see the electronic supplementary material, figures S15–S18.

Plotting levels of crurotarsan mandibular morphological and biomechanical disparity through time reveals contrasting patterns (figure 1*a*,*b*). Morphological disparity was highest in the Late Triassic before an abrupt decline into the Early, Middle and Late Jurassic. This is followed by a considerable rise in morphological disparity in the Cretaceous, with Late Cretaceous disparity levels approaching the Late Triassic maximum. In contrast to morphological disparity, biomechanical disparity shows a decrease across the TJB followed by stability through the Jurassic and Cretaceous (figure 1*b*). There is no low trough in biomechanical disparity in the Middle Jurassic and no increase during the Cretaceous.

The trend of decreasing morphological disparity into the Middle and Late Jurassic is partially supported by significantly different levels of disparity between the Late Triassic and the Middle (*p* < 0.001) and Late (*p* = 0.001) Jurassic (see the electronic supplementary material, table S13). Similarly, increasing morphological disparity from the Middle Jurassic to the high peak of the Late Cretaceous is partially reflected by statistically contrasting levels of disparity between the Middle Jurassic and the Early Cretaceous (*p* = 0.030, insignificant when corrected for multiple comparisons), and between the Late Jurassic and Late Cretaceous (*p* = 0.007) (see the electronic supplementary material, table S13). LRs for the Early–Middle Jurassic and Late Jurassic–Early Cretaceous transitions are also higher than others (3.39 and 3.66, respectively), despite not exceeding the significance threshold value of 8.0 [26] (see the electronic supplementary material, table S14). The stable levels of biomechanical disparity in the Jurassic and Cretaceous are confirmed by statistically indistinguishable variance between Jurassic and Cretaceous time bins (*p*-values ranging from 0.467 to 0.988 and LRs of 1.00–1.34) (see the electronic supplementary material, tables S15 and S16). Biomechanical disparity remains stable despite higher sample sizes in the Late Jurassic and Cretaceous (see the electronic supplementary material, table S5).

**Figure 2.**
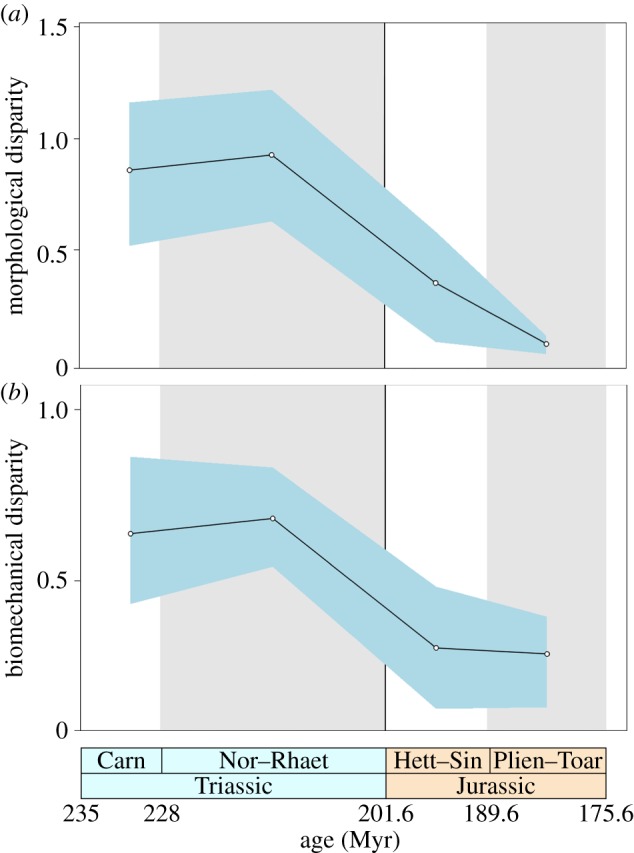
Crurotarsan mandibular morphological and biomechanical disparity across the TJB. Morphological (*a*) and biomechanical (*b*) disparity (sum of variances) are plotted in four time bins: Carnian, Norian–Rhaetian, Hettangian–Sinemurian and Pliensbachian–Toarcian. The shaded components represent 95% CIs based on 1000 bootstrap pseudoreplicates.

By examining the TJB in greater detail, it is evident that the drop in morphological and biomechanical disparity was abrupt (figure 2). Crurotarsans were a morphologically and biomechanically disparate group in both the Carnian and Norian–Rhaetian, but there was a sharp reduction in disparity by the Hettangian–Sinemurian, after the extinction of the non-crocodylomorph crurotarsans. The drop in morphological disparity remains insignificant statistically (*p* = 0.066, LR 1.70), whereas the drop in biomechanical disparity was significant (*p* = 0.016, LR 1.46, insignificant when corrected for multiple comparisons). Morphological disparity continued to decline significantly in the Pliensbachian–Toarcian (*p* = 0.044, LR 5.23, insignificant when corrected for multiple comparisons) but biomechanical disparity remained stable (*p* = 0.995, LR 1.00) (see the electronic supplementary material, table S17).

### (b) Dissecting the disparity trends

Both partial morphological disparity and partial biomechanical disparity reveal the successive dominance of three major taxonomic assemblages throughout the Mesozoic (figure 1*c*,*d*). These represent the major ecological radiations of Mesozoic crurotarsans that underlie overall patterns in morphological and biomechanical disparity.

In the Late Triassic, non-crocodylomorph crurotarsans were dominant contributors to overall disparity, with Triassic crocodylomorphs remaining largely subordinate. Sphenosuchians and ‘protosuchians’ became significant contributors to partial disparity in the Early Jurassic after the extinction of non-crocodylomorph crurotarsans. However, their relative contribution is dramatically diminished in subsequent time bins owing to a decline in relative diversity, driven by the radiations of Middle–Late Jurassic thalattosuchians and Cretaceous neosuchians and notosuchians.

Thalattosuchian crocodylomorphs were primary contributors to both morphological and biomechanical disparity in the Jurassic. Teleosaurids originated in the Early Jurassic, followed by the diversification and dominance of metriorhynchids by the Middle Jurassic. This coincides with the low trough in overall morphological disparity (figure 1*a*). Further interpretation of this pattern is limited by our restricted sampling of crocodylomorph diversity in the Jurassic, owing to a lack of material from other clades (see further below).

Cretaceous time bins are characterized by a distinct taxonomic turnover and the dominance of neosuchians and notosuchians, whereas other groups make relatively minor contributions to overall disparity (figure 1*c*,*d*). This period is associated with an overall increase in morphological disparity (figure 1*a*), whereas overall biomechanical disparity levels are only maintained (figure 1*b*). While neosuchians were diverse in the Cretaceous and achieved worldwide distribution, extensive ghost lineages trace the origins of the clade to the Early Jurassic [28], making it difficult to interpret how rapid this morphological diversification was. The fossil record of Early and Middle Jurassic neosuchians is very sparse and is unsampled in our analyses.

Trends in partial morphological disparity and partial biomechanical disparity were generally congruent in terms of the relative contribution of dietary ecologies to major macroevolutionary patterns (figure 1*e*,*f*). Coinciding with maximum disparity in the Late Triassic, representatives of all dietary groups were present, with herbivorous and piscivorous taxa contributing most to overall disparity. Through the Jurassic, piscivorous crurotarsans were dominant contributors to both disparity metrics, relating to the radiation of thalattosuchians and coinciding with reduced levels of morphological disparity, whereas biomechanical disparity did not reduce to such an extent (figure 1*a*,*b*). In our sample, herbivorous crurotarsans are not present in the Jurassic, large carnivores are missing from the Early and Middle Jurassic and small carnivores are not represented in the Middle and Late Jurassic. Increased taxonomic diversity and morphological disparity in the Cretaceous is correlated with a turnover in represented feeding ecologies and a significant reduction in the relative contribution by piscivores. Small carnivores made a large contribution to both disparity metrics in the Early Cretaceous, and large carnivores and herbivores remained major contributors in both Cretaceous bins. Despite an abundance of feeding ecologies in the Cretaceous, the amount of biomechanical variation in the jaws did not increase, but instead remained stable (figure 1*b*).

The most salient result, when grouping Mesozoic crurotarsans according to proposed mode of life and habitat, is the overwhelming contribution of marine taxa during the Jurassic, relating to dominance by piscivorous thalattosuchians in our dataset and a period of low morphological disparity (figure 1*a*,*g*,*h*). The Late Triassic and Late Cretaceous peaks in overall morphological disparity (figure 1*a*) were dominated by terrestrial and putatively fossorial taxa. Although terrestrial and fossorial taxa are diverse in both the Late Triassic and the Cretaceous, biomechanical variation is considerably lower in the Cretaceous (notosuchian dominated) than in the Late Triassic (dominated by non-crocodylomorph crurotarsans) (figure 1*b*,*h*).

### (c) The trajectories of morphological and biomechanical radiations

**Figure 3.**
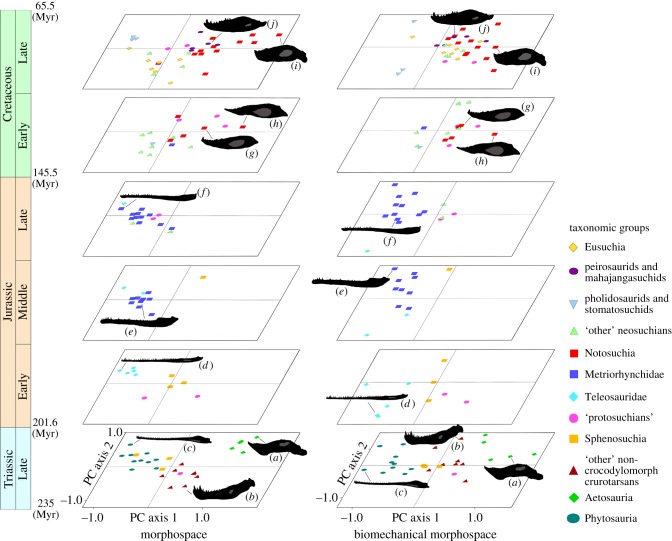
Patterns of crurotarsan morphospace and biomechanical morphospace occupancy through the Mesozoic. Taxa are plotted in six time bins: Late Triassic, Early Jurassic, Middle Jurassic, Late Jurassic, Early Cretaceous and Late Cretaceous. Plots are based on coordinate axes 1 and 2 from the principle components analysis and principle coordinates analysis (see the electronic supplementary material, tables S9–S11 and figures S13 and S14). Numerous exemplary jaws are highlighted to provide context: (*a*) *Desmatosuchus haplocerus* (Aetosauria), (*b*) *Postosuchus kirkpatricki* (other non-crocodylomorph crurotarsan), (*c*) *Mystriosuchus planirostris* (Phytosauria), (*d*) *Pelagosaurus typus* (Teleosauridae), (*e*) *Metriorhynchus superciliosus* (Metriorhynchidae), (*f*) *Cricosaurus araucanensis* (Metriorhynchidae), (*g*) *Pakasuchus kapilimai* (Notosuchia), (*h*) *Malawisuchus mwakasyungutiensis* (Notosuchia), (*i*) *Simosuchus clarki* (Notosuchia) and (*j*) *Mahajangasuchus insignis* (mahajangasuchid).

To complement the partial disparity analyses and facilitate a visual examination of morphological and biomechanical variation through time, morphospace and biomechanical morphospace were plotted in six epoch-level time bins (figure 3). Details describing the ordination axes are provided in the electronic supplementary material, tables S9–11 and figures S13 and S14. The high levels of morphological and biomechanical disparity in the Late Triassic are related to a diverse range of mandibular morphologies and biomechanical profiles, evolved by phytosaurs, aetosaurs, ‘rauisuchians’, ornithosuchids and basal crocodylomorphs (figure 3). After the extinction of non-crocodylomorph crurotarsans across the TJB, there was an overall reduction of morphospace and biomechanical morphospace occupation in the Early Jurassic. The Middle and Late Jurassic time bins were dominated by a single morphotype and biomechanical profile, exhibited by piscivorous marine thalattosuchians, localized in left and central left regions of both spaces. These taxa are characterized by highly elongate gracile mandibles with large symphyses, a higher percentage of the mandible bearing dentition and weak, rapid bites. In the Early Cretaceous, patterns of mandibular morphospace and biomechanical morphospace occupation departed considerably from previous time bins (*p* < 0.001) (see the electronic supplementary material, table S12). There was a radiation into the lower left and right quadrants of morphospace and the right quadrants of biomechanical morphospace, by notosuchians and neosuchians (figure 3). This reflects a greater variation of more robust mandibular forms and taxa possessing more powerful bites, linked to the evolution of large carnivorous and herbivorous terrestrial crocodylomorphs (figure 1*e–h*). However, despite a fundamental shift in ecological structure, biomechanical disparity remained stable (figure 1*b*). By the Late Cretaceous, crurotarsan morphospace and biomechanical morphospace had expanded to encompass most of the range occupied by Late Triassic crurotarsans, suggesting that crocodylomorphs revisited vacated crurotarsan ecological roles during this time.

## 4. Discussion

### (a) The Mesozoic crocodylomorph fossil record

The divergent patterns of morphological and biomechanical disparity following the Early Jurassic identified here (figure 1*a*,*b*) must be considered in the context of both Mesozoic crocodylomorph ecological radiations (figures 1*c–h* and 3) and the variation in sampling between the marine Jurassic record and largely terrestrial Cretaceous record. Middle and Late Jurassic crocodylomorphs have a poor terrestrial record, owing to a reduction in terrestrial fossiliferous units and outcrop area linked to marine transgressions [29]. However, the fossil record of marine Jurassic thalattosuchians is very rich and fossils are preserved in high abundance [6,8,30]. By contrast, Cretaceous crocodylomorphs are relatively well represented globally [31], with terrestrial neosuchians achieving a worldwide distribution by the Early Cretaceous. However, the earliest known neosuchian, *Calsoyasuchus valliceps*, is from the Early Jurassic, placing the origins of the clade around 50 Myr before they become abundant in the fossil record [28]. This ghost range implies that the Mesozoic crocodylomorph fossil record is punctuated by a major gap in the Middle and Late Jurassic. Our results are therefore interpreted as representing three distinct evolutionary events, during which the dynamics of morphological and biomechanical evolution vary: (i) the initial radiation of ecologically diverse Late Triassic crurotarsans, associated with exceptionally high levels of mandibular morphological and biomechanical variation; (ii) the radiation of specialized Jurassic marine crocodylomorphs, linked to reduced mandibular morphological variation and moderate biomechanical disparity; and (iii) the Cretaceous diversification of terrestrial crocodylomorphs, during which there was a large increase in mandibular morphological variation but no apparent increase in biomechanical variation.

### (b) Ecological diversity of Late Triassic crurotarsans

A diverse range of crurotarsan archosaurs dominated Late Triassic terrestrial ecosystems approximately 20 Myr after the Permian–Triassic extinction [32]. Our disparity metrics indicate that Late Triassic crurotarsans evolved a large range of both mandibular morphologies and biomechanical profiles (figures 1*a*,*b*, 2 and 3). These include elongate, gracile jaws with weak rapid bites and scissor-like occlusion in the fish and flesh-eating phytosaurs and more robust blunted jaws, with slow powerful bites, characteristic of large carnivores and armoured herbivorous aetosaurs. This corroborates other studies that used alternative proxies to conclude that crurotarsan ecological diversity was high in the Late Triassic [2,9,10]. We discover that crurotarsans suffered a major perturbation across the TJB, with a decline in both mandibular morphological and biomechanical variation (figure 2). This supports conclusions from cladistic disparity analyses based on whole body characters in Late Triassic and Early Jurassic crurotarsans [9]. However, it conflicts with patterns observed in a recent study based on variation in cranial characters only, where no change in morphological disparity was observed across the TJB [10]. The discrepancy between our study and [10] cannot be attributed to variations in sampling, as both analyses have a dataset composed of similar taxa. Instead, it probably relates to contrasting methods of quantifying variation [13]. Our analyses focus on variation in mandibular form and biomechanical function, which has fundamental significance to feeding, whereas cladistic characters derived from phylogenetics are originally designed to establish evolutionary relationships and differentiate clades, most without any particular relevance to feeding ecology or biomechanics.

### (c) The radiation of Jurassic marine crocodylomorphs

The trophic radiation of thalattosuchians during the Jurassic may be related to marine transgressions, providing more epicontinental marine habitats [33]. This ecospace was exploited by crocodylomorphs, which evolved a specialized marine piscivorous ecological role associated with a restricted mandibular form and distinct biomechanical characteristics (figure 3).

The thalattosuchian mandibular morphotype is generally constrained to a highly elongate and dorsoventrally flattened form, that facilitated medio-lateral excursions and minimized pressure drag during lateral sweeps of the jaw, aiding capture of fast fleshy prey [34–36] (see the electronic supplementary material, figure S13). The structural constraints on this form appear to have led to low levels of morphological disparity in the Jurassic (figure 1*a*). Dynamics of an aquatic medium and a piscivorous diet have been shown to have profound effects on the shape of skulls and lower jaws in turtles, sauropterygians and modern and extinct crocodylomorphs [34–38]. Metriorhynchid thalattosuchians evolved a hypercarnivorous marine ecology [6], that is associated with a more robust mandibular form, but it remained confined to central morphospace (figure 3, Late Jurassic/Early Cretaceous).

Marine piscivorous thalattosuchians also had distinct biomechanical adaptive features for capturing fast-moving fleshy prey, including large mandibular symphyses that reduce stress during rapid movements, a high percentage dentition increasing the area of the jaw available for prey capture, and low opening and closing mechanical advantages, producing weak rapid bites [14,39] (see the electronic supplementary material, figure S14). While morphological disparity declined through the Early and Middle Jurassic, biomechanical disparity in marine piscivorous thalattosuchians remained stable. Presumably, biomechanical changes to the thalattosuchian mandible allowed morphologically similar taxa to feed on varying food resources [38]. Evolving biomechanical variation that did not modify lateral jaw morphology may have encouraged phenotypic variation that avoided compromising hydrodynamic efficiency.

### (d) Cretaceous diversification of terrestrial crocodylomorphs

Unlike the thalattosuchian radiation in the Jurassic, the Cretaceous trophic radiation of crocodylomorphs took place primarily in the terrestrial realm (figure 1*g*,*h*) [31,40]. In the Early Cretaceous, semiaquatic neosuchians diversified and numerous terrestrial ‘protosuchians’ and notosuchians became abundant in the fossil record. By the Late Cretaceous, morphospace and biomechanical morphospace occupation expanded into areas previously vacant or scarcely explored since the Late Triassic (figure 3). Notosuchians, famed for disparate cranial and postcranial morphologies, evolved an array of ‘mammal-like’ mandibular forms and varied biomechanical profiles, associated with herbivorous, small carnivorous and large carnivorous ecologies (figures 1*c–f* and 3). It is intriguing that our study returns expected high levels of morphological disparity for mandibular elements during the Cretaceous, but total biomechanical disparity remains unchanged, despite the evolution of this exceptional group and multiple others.

The absence of an increase in biomechanical disparity during the Cretaceous, in light of increased morphological evolution, could have arisen in several ways. The lack of constraints imposed by an aquatic medium may have resulted in more morphological variation in terrestrial taxa. This would explain why peaks in morphological disparity in the Late Triassic and Cretaceous coincide with dominance of terrestrial (and fossorial) crurotarsans (figure 1*a*,*g*). Alternatively, it is possible that biomechanical evolution in Cretaceous groups, particularly the notosuchians, was concentrated to other areas of their anatomy. This is supported by the appearance of novel postcranial morpho-functional innovations (e.g. *Armadillosuchus* and *Simosuchus*) and the widespread evolution of heterodonty and bizarre ‘mammal-like’ dentitions (e.g. *Pakasuchus* and *Mariliasuchus*) [3,7,41–43]. Indeed, such features may have made significant biomechanical mandibular evolution have less adaptive value. Morphological evolution may have continued despite biomechanical stability to improve the flexibility of design, allowing forms to evolve secondary functions, without compromising their primary function [15].

The lack of mandibular biomechanical disparity in the Cretaceous does not detract from the exceptional nature of the crocodylomorph radiation during this period. Both notosuchians (baurusuchids) and mahajangasuchids convergently evolved a terrestrial hypercarnivorous ecology, associated with a robust mandibular form, high jaw opening and closing mechanical advantages and increased resistance to bending stresses (figure 3 and the electronic supplementary material, figures S13 and S14). The evolution of large terrestrial hypercarnivorous crocodylomorphs, comparable to the ‘rauisuchians’ of the Late Triassic, suggests that in some parts of a dinosaur-dominated world, crocodylomorphs were able to compete as apex terrestrial predators [44]. Additionally, notosuchians evolved a number of herbivorous ecomorphological indicators present in aetosaurs, lizards, dinosaurs and mammals [3,7,43,45,46]. These include robust jaws with large mandibular fenestrae to accommodate increased jaw musculature, high mechanical advantages of jaw opening and closing providing slow but powerful bites, and large quadrate offsets resulting in simultaneous contact of the dentition; all biomechanical traits that improve the processing of plant matter (figure 3 and the electronic supplementary material, figures S13 and S14). Perhaps the rarity of mammalian taxa in Gondwana during the Cretaceous facilitated the adaptive radiation of such crocodylomorphs into this distinctive vacant ecospace [3,47,48].

## 5. Conclusion

The decoupling of morphological and biomechanical disparity demonstrated here has been identified in other studies of both extant and extinct taxa [14,15,18], revealing how the application of both metrics can provide multifaceted insights into the evolution of feeding systems. The lack of correlation between morphological and biomechanical disparity during the evolution of Mesozoic crocodylomorph lower jaws can be attributed to two contrasting radiations, where dietary ecology and habitat variably acted as both constraints and stimuli for morphological and biomechanical evolution. Overall, the evolution of non-carnivorous dietary strategies appears to have enabled crurotarsans to explore a more diverse range of morphologies and biomechanical characteristics, beyond the limitation of a carnivorous ancestral ecology. A similar trend has been reported in theropod dinosaurs, where dietary plasticity has been postulated to facilitate morphological and biomechanical evolution [19,46]. Regardless of conflicting trends between morphological and biomechanical disparity, the radiation of crocodylomorph crurotarsans following the ETE remains truly exceptional, as a single clade went on to reoccupy varied ecological niches despite significant competition in both the marine (sauropterygians and ichthyosaurs) and terrestrial (dinosaurs and mammals) realms.

## Data accessibility

All datasets are deposited in the Dryad repository: http://dx.doi.org/10.5061/dryad.61s1n.
